# The role of diffusion-driven pure climb creep on the rheology of bridgmanite under lower mantle conditions

**DOI:** 10.1038/s41598-018-38449-8

**Published:** 2019-02-14

**Authors:** Riccardo Reali, James A. Van Orman, Jeffrey S. Pigott, Jennifer M. Jackson, Francesca Boioli, Philippe Carrez, Patrick Cordier

**Affiliations:** 1 University Lille, CNRS, INRA, ENSCL, UMR 8207 − UMET − Unité Matériaux et Transformations, Lille, F-59000 France; 2 Department of Earth, Environmental, and Planetary Sciences − Case Western Reserve University, 10900 Euclid Avenue, Cleveland, OH 44106 USA; 3 Present Address: Shock and Detonation Physics (M-9), Los Alamos National Laboratory, Los Alamos, NM 87545 USA; 4 Seismological Laboratory, Division of Geological and Planetary Sciences − California Institute of Technology, Pasadena, CA 91125 USA; 5 Present Address: LEM, UMR 104 CNRS/ONERA, Chatillon, 92320 France

## Abstract

The viscosity of Earth’s lower mantle is poorly constrained due to the lack of knowledge on some fundamental variables that affect the deformation behaviour of its main mineral phases. This study focuses on bridgmanite, the main lower mantle constituent, and assesses its rheology by developing an approach based on mineral physics. Following and revising the recent advances in this field, pure climb creep controlled by diffusion is identified as the key mechanism driving deformation in bridgmanite. The strain rates of this phase under lower mantle pressures, temperatures and stresses are thus calculated by constraining diffusion and implementing a creep theoretical model. The viscosity of MgSiO_3_ bridgmanite resulting from pure climb creep is consequently evaluated and compared with the viscosity profiles available from the literature. We show that the inferred variability of viscosity in these profiles can be fully accounted for with the chosen variables of our calculation, *e.g*., diffusion coefficients, vacancy concentrations and applied stresses. A refinement of these variables is advocated in order to further constrain viscosity and match the observables.

## Introduction

Significant insight into the major features that affect the surface of the Earth (seismicity, volcanism, mountain building) has been gained through the understanding that global convection animates the mantle to dissipate the internal heat of our planet. However, some fundamental aspects of mantle dynamics remain poorly understood, including the rheology.

From the observational point of view, the first constraint on the rheology of the mantle was deduced from the analysis of post−glacial uplift^1^, which assessed a uniform viscosity of the mantle to be ~10^21^ Pa·s. This estimation has proved to be robust over the years. However, further studies based on modelling of mantle convection^2,3^, the geoid^4–7^, tectonic plate velocities^8,9^, true polar wander^10^ and length of day variations^11^ have consistently pointed out a significant increase in viscosity (10^1^–10^2^ times) between the upper and the lower mantle. Most proposed profiles consist of a broad viscosity hill in the middle of the mantle, at a depth roughly between 1200 and 2000 km^12–15^. Some recent studies, however, suggest a rheology contrast located around 1000 km depth^16,17^.

Another strong constraint on deep mantle convection is linked to seismic anisotropy, which can provide clues on the active deformation mechanisms. Very early, dislocation creep was postulated as a potential deformation mechanism in crystalline rocks in the mantle^18^. Besides implying a non−linear rheology, dislocation creep is also very efficient in producing crystal preferred orientation (CPO) and this is a very fruitful line of interpretation of seismic anisotropy in the upper mantle^19^. In a thick layer consisting of elastically anisotropic phases, such as the lower mantle, activation of dislocation creep is expected to generate detectable seismic anisotropy, which is inconsistent with observations^20^. Although most of the lower mantle is relatively under−sampled in terms of seismicity, the absence of strong evidence for seismic anisotropy between 700 km and 2700 km^19,21^ provides some constraints. This lack of anisotropy has led to the common assumption that diffusion creep, which does not lead to CPO, is the dominant deformation mechanism in the lower mantle^22^. Unlike dislocation creep, diffusion creep corresponds to a linear viscous behaviour. Most importantly, diffusion creep is strongly grain size−dependent. Since this parameter is extremely poorly constrained in the mantle, the conditions imposed by the mean grain size, the grain size distribution and its possible evolution have attracted attention recently^23,24^.

Mineral physics represents another important approach to place constraints on mantle convection. Bridgmanite, *i.e*. (Mg,Fe,Al)(Si,Fe,Al)O_3_ with the orthorhombic perovskite structure, is thought to be elastically anisotropic^25^ and is considered to be the main constituent of the bulk lower mantle, along with (Mg,Fe)O ferropericlase and CaSiO_3_ perovskite^26^. The rheology of this mineral is thus of primary importance to understand and model convection in the mantle and the dynamics of Earth’s interior. However, bridgmanite is only stable under lower mantle conditions and measurements of the strength of materials under simultaneous high pressures and high temperatures are extremely difficult. Multiple studies have been devoted to creep in minerals with the perovskite structure (e.g. BaTiO_3_^27^, KTaO_3_ and KNbO_3_^28^, CaTiO_3_ and NaNbO_3_^29^, SrTiO_3_^30^), providing evidence for dislocation activity. As for bridgmanite, the first deformation experiments performed at room temperature in the diamond anvil cell (DAC) suggested a high strength with little if any indication of plasticity by dislocation glide^31–33^. More recent experiments using laser−heated DAC showed, however, some indication of plastic slip in bridgmanite^34^. The development of opposed− and multi−anvil devices to allow high pressure and high temperature deformation experiments represents a major breakthrough, since it opens the path for direct investigation of mantle phases^35^. Using the recently developed rotational Drickamer apparatus (RDA), the first deformation experiments on a bridgmanite and ferropericlase assemblage at pressures and temperatures of the uppermost lower mantle have been performed^36^. It was demonstrated that even at a temperature in excess of 2000 K, bridgmanite retains a very high strength (~4–5 GPa). Microstructural investigation revealed that bridgmanite deformed under these conditions through activation of dense shear lamellae^37^. In another study where bridgmanite was deformed at 25 GPa, 1873 K in a deformation-DIA apparatus^38^, a clear development of crystal preferred orientation was linked to the activation of [001](100) slip. No stress was reported in this study. Some studies have, however, demonstrated the possibility for diffusion creep and even superplasticity in CaTiO_3_, a mechanism which could be compatible with seismic observations^39–41^.

In parallel, recent theoretical calculations related to the physics of dislocations in MgSiO_3_ bridgmanite confirm that the lattice friction remains very high, even at high temperature^42^, which account very well for the high stress levels observed in experiments^36^. Indeed, insights into slip systems and plastic anisotropy of MgSiO_3_ bridgmanite were first gained from modelling dislocation cores and lattice friction at the atomic scale^43–47^. More recently, high temperature dislocation glide has been characterized from the modelling of thermally activated processes underlying dislocation glide at finite temperature^42^. From these theoretical developments, the velocity of dislocations and the flow stress of the material could be calculated not only at pressures and temperatures relevant to the lower mantle, but also at relevant strain rates, without extrapolation or parameter adjustment. For MgSiO_3_ bridgmanite, such calculations demonstrate that lattice friction remains very high, even at high temperatures and mantle strain rates, and that deformation through dislocation glide appears to be extremely unfavourable at mantle conditions^48^.

However, glide is not the only possible mechanism for dislocations to move and produce strain. At high temperature, dislocations in bridgmanite interact strongly with vacancies and, by absorbing them, can move via climb^49^. Deformation by climb, without glide, and possibilities for creep involving it, has received little attention, although it had been originally suggested that it might be relevant for rheology in planetary interiors^50^. Some microstructural evidence for climb has been reported, however, in KTaO_3_ and KNbO_3_ perovskite deformed by creep at high temperature^28^. Furthermore, it has been demonstrated by dislocation dynamics modelling that dislocations in bridgmanite moving by climb alone could lead to steady state creep with a strain−producing efficiency superior to that of diffusion creep^51^. The main implication of this discovery is that the poorly constrained grain size is not a factor controlling the rheology of bridgmanite in the uppermost lower mantle. Although pure climb creep is mediated by dislocation motion, it is similar to diffusion creep, for which strain is diffusion−controlled. As with diffusion creep, pure climb creep is consistent with the observed isotropy in the bulk of the lower mantle, since materials deforming by this mechanism do not develop rigid body rotations, which are responsible for CPO. Diffusion, therefore, represents the key to the rheology of bridgmanite. In the present paper, we revisit the available data on diffusion in bridgmanite under lower mantle conditions. The results are incorporated into a pure climb creep model^50^ to propose constraints on the viscosity of bridgmanite in the lower mantle.

## Diffusion in Bridgmanite

The rate of dislocation climb is ultimately limited by self−diffusion of the slowest diffusing atomic species. Experimental studies of self−diffusion in bridgmanite indicate that oxygen^52,53^ diffuses significantly faster than Mg or Si^53–55^. This feature is shared with many other perovskite−structured oxides, in which the energy of oxygen vacancy migration is more favourable compared to the one of cation vacancies, leading to rapid oxygen transport and sluggish cation diffusion^56^. We therefore focus on cation diffusion as the rate−limiting step for dislocation climb in bridgmanite.

Self−diffusion coefficients for Mg and Si^53–55^ have been measured at pressures relevant to the top of the lower mantle (~25 GPa) using a multi−anvil press combined with secondary ion mass spectrometry (SIMS) in depth profiling mode. Both polycrystalline^53,54^ and single crystal^55^ bridgmanite samples were tested. In the former case, both volume and grain boundary diffusion coefficients were extracted, whereas in the latter the single crystals avoided complications from grain boundary diffusion. In all cases, however, the characteristic volume diffusion length was < 200 nm. The interpretation of such short profiles from high−pressure experiments is uncertain, due to analytical artefacts that arise from surface roughening and various ion beam mixing effects in SIMS depth profiling. To have a high level of confidence in the diffusion coefficient measurements, particularly when the diffusion profiles are so short, it is critical to measure the diffusion coefficient in separate experiments at the same conditions, over a range of time that is sufficient to generate substantially different profile lengths. Ideally, the diffusion anneal times at one set of conditions would extend over at least an order of magnitude, which would generate diffusion profiles that range in length over approximately a factor of 3. None of the experimental self−diffusion studies on bridgmanite have included a convincing time series – the range of times extends only over a factor of ~2, and there is no resolvable change in the length of the profiles with time. Because we cannot be confident that the cation isotope concentration profiles presented in any of the published studies represent true volume diffusion profiles, we conservatively treat the diffusion coefficients extracted from these profiles as upper bounds on the true diffusion coefficients. We focus below on the single crystal experiments^55^, because they characterized both silicon and magnesium diffusion profiles, but these data agree within uncertainty with the results obtained with polycrystals^53,54^.

Further constraints on cation diffusion coefficients in bridgmanite come from first principles calculations and atomistic simulations. Theoretical calculations of the self−diffusion coefficients (*D*^*sd*^) are based on the following equation:

1 $${D}^{sd}=f{X}_{v}{D}_{v}\,$$

where *f*, the correlation coefficient, has a value close to unity and is often neglected, *X*_*v*_ is the fraction of vacant sites on the relevant sub−lattice, and the vacancy diffusion coefficient, *D*_*v*_, is:

2 $${D}_{v}=\frac{Z}{6}{l}^{2}\nu \,\exp (-\frac{{\rm{\Delta }}{H}_{m}}{{k}_{B}T})$$

where *Z* is the number of equivalent jumps that a vacancy can make to adjacent sites, *l* is the jump distance, *ν* is the jump attempt (or vibration) frequency, *ΔH*_*m*_ is the migration enthalpy, *k*_*B*_ is the Boltzmann constant, and *T* is the temperature in Kelvins. The migration enthalpies, jump distances, and vibration frequencies for Mg and Si have been obtained by density functional theory calculations over the pressure range of the lower mantle^57,58^. The migration enthalpies calculated from first principles, using the local density approximation, are equivalent for Si (3.6 eV) and Mg (3.4–3.7 eV) at 25 GPa^58^, consistent with the apparent similarity in Si and Mg diffusion coefficients. We use the values for Mg, because a more extensive set is available^57,58^ than for Si^58^, and because Mg diffuses by a simple direct jump process, rather than the complicated six−jump cycle used by Si. The migration enthalpy for magnesium as a function of pressure is based on simulations of Mg diffusing along the lowest energy pathways ([110]a,b and [001]) via a single jump^57^, resulting in *Z* = 6 possible jumps (two equivalent jumps for each principal direction). The jump distances and frequencies as functions of pressure are from Ammann *et al*.^58^. The self−diffusion coefficients can then be calculated according to equation (1), provided that the vacancy concentration is known.

The vacancy concentration can be bounded at the low end by the intrinsic concentration of vacancies, which depends only on the vacancy formation energy. Atomistic simulations show that the SiO_2_ pseudo−Schottky defect is the least favourable defect and therefore that Si is the rate−limiting species in an intrinsic diffusion regime^59^. Assuming a purely intrinsic defect mechanism, we calculate the Si vacancy concentration as a function of pressure based on this SiO_2_ pseudo−Schottky formation enthalpy from Watson *et al*.^59^. This results in an intrinsic Si vacancy concentration of 10^−20^–10^−14^ throughout the pressure range of the lower mantle. At the high end, we can define the upper limits on the diffusion coefficient considering an extrinsic regime as determined by experiments, with an evaluation of a reasonable interval of variability for the vacancy concentration.

It is possible to indirectly estimate an upper limit on *X*_*v*_ from equation (1) by combining the *D*^*sd*^ coefficients obtained by Xu *et al*.^55^ on magnesium self−diffusion in bridgmanite and the *D*_*v*_ values estimated using the Ammann *et al*.^58^ approach at the same pressure and temperatures. In Table 1 the numerical values for *D*^*sd*^ and *D*_*v*_ from experiments and first principles atomistic simulations, respectively, are provided together with the *X*_*v*_ values, estimated using equation (1). The pressure is here fixed at 25 GPa. This calculation gives upper limits on the Mg vacancy concentration that are consistently on the order of 10^−2^. If the vacancy concentration were higher and diffusion faster, the measured profiles would have been longer. Hence, we treat 10^−2^ as the upper bound on the cation vacancy concentration in bridgmanite in these experiments and by extension, the lower mantle. Although this upper bound estimate was obtained from experiments on nominally pure MgSiO_3_, we note that the addition of trace or minor elements is unlikely to change the upper bound on the rate-limiting vacancy concentration significantly. If trivalent cations partition preferentially onto the Si site, there will be an increased production of extrinsic oxygen vacancies, suppressing the concentration of cation vacancies. If instead trivalent impurities partitioned preferentially onto the Mg site in lower mantle bridgmanite, this would enhance the cation vacancy concentrations. However, this type of disorder would also depress oxygen vacancy concentrations to extremely low values (at the intrinsic level, or below). Diffusion of oxygen would then be rate-limiting for climb, and would be quite sluggish with vacancy concentrations many orders of magnitude below 10^−2^.

**Table 1:** The vacancy concentration *X*_*v*_ is indirectly estimated using equation (1). The self−diffusion coefficients *D*^*sd*^ are from Xu *et al*.^55^ and the vacancy diffusion coefficients *D*_*v*_ are calculated from Ammann *et al*.^58^. Both *D*^*sd*^ and *D*_*v*_ are estimated at 25 GPa.

*T* (K)	*D*^*sd*^ (m^2^/s)^55^	*D*_*v*_ (m^2^/s)^58^	*X*_*v*_
1670	2.6 ∙ 10^−20^	2.6 ∙ 10^−18^	1.0 ∙ 10^−2^
1770	1.5 ∙ 10^−20^	1.1 ∙ 10^−17^	1.4 ∙ 10^−3^
1870	2.1 ∙ 10^−19^	4.0 ∙ 10^−17^	5.2 ∙ 10^−3^
1870	1.9 ∙ 10^−19^	4.0 ∙ 10^−17^	4.6 ∙ 10^−3^
2070	1.1 ∙ 10^−18^	3.6 ∙ 10^−16^	2.9 ∙ 10^−3^

**Figure 1:**
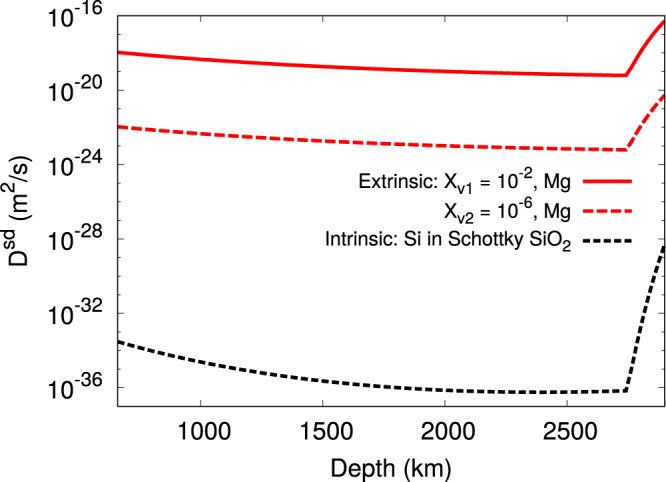
Diffusion coefficients *D*^*sd*^ along the geotherm. Here are shown the values for intrinsic diffusion obtained from the Si Schottky vacancy formation enthalpy of Watson *et al*.^59^ (black dashed line) together with the extrinsic values for Mg obtained with the selected vacancies concentrations *X*_*v1*_ and *X*_*v2*_ (solid and dashed red lines, respectively).

In Table 2 all the necessary parameters to calculate the self–diffusion coefficients at different pressures are provided. In Fig. 1 we show the diffusion coefficients calculated according to equations (1) and (2) along a mantle geotherm^60^. It is evident that the values calculated considering an intrinsic vacancy concentration for Si are not meaningful with respect to terrestrial time scales of deformation. Hence, we consider an upper bound of 10^−2^ for the rate-limiting vacancy concentration, and also consider, for the purposes of illustration, a much lower vacancy concentration of 10^−6^ as inputs in the creep model for bridgmanite, which is described in the next paragraph.

**Table 2:** Estimate of the parameters necessary to evaluate the self–diffusion coefficients *D*^*sd*^ at different pressures along the geotherm^60^ using equations (1) and (2). The vacancy concentration *X*_*v*_ is here set equal to 10^−2^.

*P* (GPa)	*Depth* (km)	*T* (K)	*ν* (THz)	*l* (Å)	*ΔH*_*Mg*_ (eV)	*D*^*sd*^ (m^2^/s)
30	809	1992	5.9	2.42	3.8	7.1 ∙ 10^−19^
60	1457	2256	8.9	2.36	4.7	2.0 ∙ 10^−19^
90	2060	2487	12.9	2.31	5.4	9.6 ∙ 10^−20^
120	2617	2689	17.5	2.27	5.9	6.5 ∙ 10^−20^

## Creep Model

In this paper, following the work of Boioli *et al*.^51^, we investigate the steady state creep behaviour of bridgmanite resulting from deformation by pure climb^50^. This mechanism, proposed by Nabarro in 1967, considers crystals containing a network of dislocations (dislocation density *ρ*) which move by pure climb (*i.e*. a displacement perpendicular to the glide plane by emission/absorption of point defects). In the original work of Nabarro^50^, the need for climb arose from a large density of jogs on the dislocation lines resulting from the mutual crossing of the dislocations, which drastically reduces the efficiency of glide. In the present case, however, climb requires attention due to the high lattice friction which inhibits glide in bridgmanite^42,48^. In pure climb creep, dislocations play the role of sources and sinks of point defects (the same role played by grain boundaries in Nabarro−Herring creep). By absorbing or emitting vacancies or ions, dislocations move by climb and produce strain (without producing lattice rotation). The strain rate resulting from this process reads^50^:

3 $$\dot{\varepsilon }=\frac{{D}^{sd}b{\sigma }^{3}}{\pi {k}_{B}T{\mu }^{2}}/ln(\frac{4\mu }{\pi \sigma })$$

where *D*^*sd*^ is the self–diffusion coefficient as discussed above, *b* is the Burgers vector, *σ* is the applied, deviatoric stress and *μ* is the shear modulus. Compared to the Boioli *et al*.^51^ simulations, where the initial dislocation density was a simulation parameter, Nabarro’s model accounts for steady state conditions by allowing the microstructure to adjust its equilibrium dislocation density with respect to the applied stress (more insights are provided in the Supplementary material). According to the derivation made by Nabarro, pure climb creep results in a non−linear rheology, where the strain rate depends on stress to the power of 3. Indeed, in pure climb creep, the dislocation network is established under the balancing influences of dipole annihilation and multiplication from the operation of Bardeen−Herring sources^61^. Despite this difference, the results of the dislocation dynamics model^51^ are in excellent agreement with the theoretical predictions of equation (3) from Nabarro^50^ (see Supplementary Fig. 1).

## Results

The pure climb creep model of Nabarro^50^ allows the calculation of the strain rates of bridgmanite at given pressures and temperatures and as a function of stress. Here we make calculations along a geotherm^60^. How the deviatoric stresses *σ* vary in the mantle is very poorly known since observables at the surface of the Earth are only related to strain or strain rate. The values most commonly accepted are of the order of a few MPa^62,63^. Since the strain rate of pure climb creep is quite sensitive to stress (stress exponent of 3), we have run calculations with deviatoric stresses in the range 1–10 MPa to account at best for the variability of this important parameter. The shear modulus *µ* of bridgmanite and its pressure−depth dependence were estimated using values from the Preliminary Reference Earth Model, PREM^64^.

**Figure 2:**
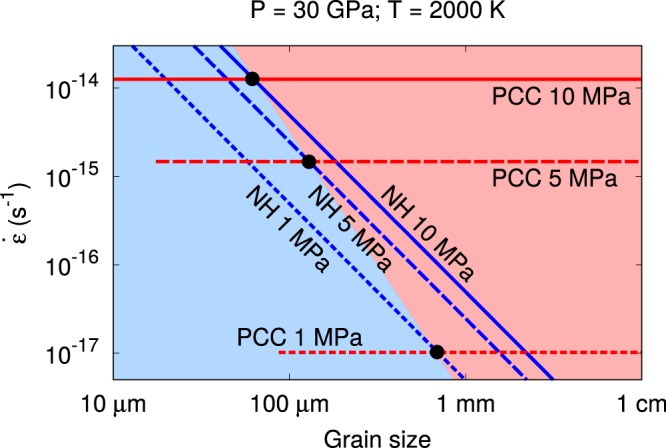
Deformation mechanism map (strain rate $\dot{\varepsilon }$ vs. grain size) at 30 GPa, 2000 K comparing pure climb creep (PCC) and Nabarro–Herring (NH) mechanisms. PCC dominates in the region marked in red and NH dominates in the blue region.

One can compare the efficiency of pure climb creep and diffusion (Nabarro–Herring) creep by building deformation mechanism maps. Here the critical parameter is the grain size. In Fig. 2, a map describing the strain rate for both mechanisms as a function of the grain size is presented. Three stresses (1, 5 and 10 MPa) are considered at given *PT* conditions along the geotherm^60^ (here 30 GPa and 2000 K, corresponding to the upper part of the lower mantle. Similar maps at 75 GPa, 2400 K and 120 GPa, 2700 K are presented in the supplementary materials). For each stress, the pure climb creep is represented as a horizontal line since this mechanism is grain–size independent. These lines exhibit a lower bound because of the existence of a critical stress needed to activate a dislocation source. In climb, dislocation multiplication is made possible by the activation of sources known as Bardeen–Herring sources^61^ which are the equivalent for climb as the Frank–Read sources observed in glide. The physics for the opening of both sources, based on the line tension, is equivalent, leading to a critical stress ${\sigma }_{c}$ for opening in the form of:

4 $${\sigma }_{c}=\frac{\mu b}{{l}_{c}}.$$

where ${l}_{c}$ is the critical size of the source. Since, for geometrical reasons, ${l}_{c}$ has to be smaller than the grain size, a bound is placed on the pure climb creep regime for a given stress.

Diffusion creep or Nabarro–Herring creep, on the other hand, is expressed by a constitutive equation which reads:

5 $$\dot{\varepsilon }={A}_{NH}\frac{{D}^{sd}\sigma {\rm{\Omega }}}{{d}^{2}{k}_{B}T}.$$

where *d* is the grain size and $\Omega$ is the atomic volume, equal to the elementary cell volume divided by the number of formula units per unit cell. The cell volume at the considered *PT* conditions was estimated from the Mie–Grüneisen–Debye Vinet equation of state for MgSiO_3_ bridgmanite^65^. ${A}_{NH}$ is a numerical factor depending on the shape of the grain and the boundary conditions, and in the impossibility of grain boundary sliding at constant stress is equal to $16/3$.

The interception of the Nabarro–Herring and pure climb creep curves for the same value of applied stress is marked by a black dot In Fig. 2. This point identifies the value of grain size for which pure climb creep becomes more efficient than Nabarro–Herring creep, *i.e*., produces a larger strain rate at the same applied stress. By connecting the different dots marking this transition at different stresses, it is possible to separate the space in different grain size domains where one mechanism is more efficient than the other. As a result, one can see that above a grain size of 0.1–1 mm, pure climb creep is expected to be the dominant mechanism.

**Figure 3:**
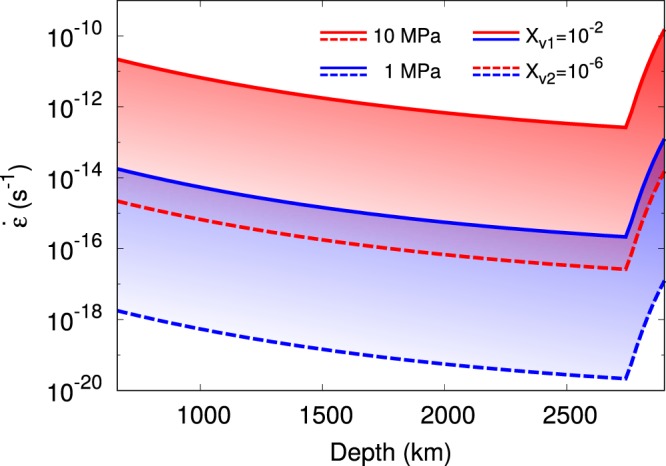
Strain rates of bridgmanite along the geotherm calculated with the Nabarro^50^ creep model for stresses of 10 (red lines) and 1 (blue lines) MPa and vacancy concentrations of 10^−2^ (solid lines) and 10^−6^ (dashed lines).

Figure 3 shows the strain rates as a function of depth considering extrinsic diffusion of Mg as the rate−limiting factor for climb. The vacancy concentrations *X*_*v1*_ and *X*_*v2*_, as discussed above, are set to 10^−2^ and 10^−6^, respectively.

Knowing the strain rates it is possible to calculate the effective viscosity *η*:

6 $$\eta =\frac{\sigma }{\dot{\varepsilon }}.$$

**Figure 4:**
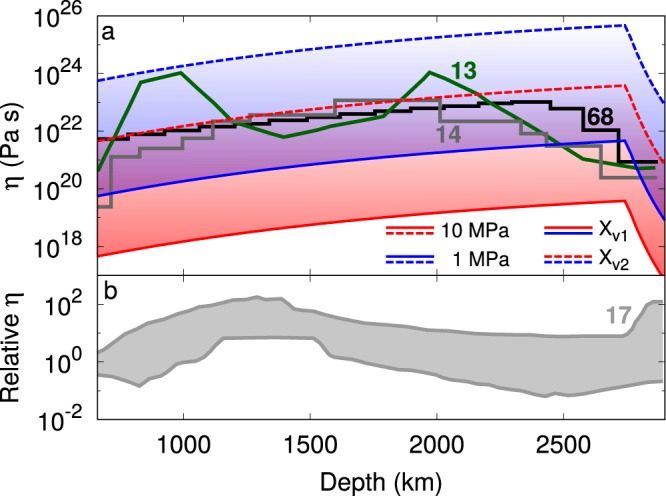
(**a**) Viscosity of bridgmanite along the geotherm for stresses of 10 (red lines) and 1 (blue lines) MPa and vacancy concentrations *X*_*v*_ of 10^−2^ (solid lines) and 10^−6^ (dashed lines). The obtained absolute values and variability of viscosity are compared with the data of Forte and Mitrovica^13^, Mitrovica & Forte^14^, Steinberger & Calderwood^68^ and (**b)** Rudolph *et al*.^17^ (R15). The scale on the y axis is the same for the (**a**) and (**b**) figures.

In Fig. 4 the calculated viscosity is plotted along the geotherm for the same stresses and vacancy concentrations described above. Figures 3 and 4 show consistently a steady, monotonic evolution of strain rate and viscosity along the geotherm^60^ until the marked temperature increase in the boundary layer at the *D*″ leads to a strong drop (ca. 3 log units) of the viscosity of bridgmanite. This simply illustrates the effect of temperature in a thermal boundary layer. Seismic observations show that this thermal boundary layer is characterized by strong lateral variations, which have been associated with rich thermochemical complexity^66,67^ and should be addressed in future studies. Our focus is on the bulk of the lower mantle, where the conjugate effect of pressure and temperature along the geotherm is relatively modest, leading to an increase of 2 log units from the base of the transition zone to the *D*″ layer. In comparison, the variability permitted by the range of stresses (1–10 MPa) and vacancy concentrations (10^−6^–10^−2^) is much larger: around 5 orders of magnitude. Table 3 shows the obtained values of strain rate $\dot{\varepsilon }$ and viscosity *η* at different pressures and temperatures considering a vacancy concentration *X*_*v*_ of 10^−2^ and an applied stress *σ* of 1 MPa (see the Supplementary material for values at different *X*_*v*_ and applied stress).

**Table 3:** Estimate of the strain rates $\dot{\varepsilon }$ and viscosities *η* at different pressures along the geotherm using the creep model of Nabarro^50^ displayed in equation (3). Here the vacancy concentration *X*_*v*_ and the deviatoric stress are set equal to 10^−2^ and 1 MPa, respectively.

*P* (GPa)	*Depth* (km)	*T* (K)	$\dot{\varepsilon }$ (s^−1^)	*η* (Pa∙s)
30	809	1992	1.0 ∙ 10^−14^	9.8 ∙ 10^19^
60	1457	2256	1.6 ∙ 10^−15^	6.3 ∙ 10^20^
90	2060	2487	5.1 ∙ 10^−16^	2.0 ∙ 10^21^
120	2617	2689	2.4 ∙ 10^−16^	4.1 ∙ 10^21^

## Discussion

It is thus interesting to compare our predictions with some viscosity profiles from the literature. In Fig. 4a, comparison is made with the models of Mitrovica & Forte^14^. It is striking that the monotonic increase of viscosity reported by Mitrovica & Forte^14^ mimics very well our prediction down to ca. 2000 km depth. The values are also in very good agreement, and it must be noted that despite the wide range of our calculations, they are quite consistent with the viscosity profile of the lower mantle suggested by most geodynamic observables. In fact, a comparison with the data of Steinberger & Calderwood^68^ confirms this agreement. The depth−dependent viscosity they obtain overlaps with the data of this study over the whole depth range of the lower mantle. The viscosity decrease reported by Mitrovica & Forte^14^ below 2000 km depth cannot be explained by our model if stress and point defect chemistry remain constant. However, the range of changes can very well be explained within reasonable variations of these parameters as considered in this study. Considering also the profile obtained by Forte & Mitrovica^13^, it is possible to conclude that the present approach brackets the existing profiles also in terms of overall variability. Although they propose a very different view of the viscosity structure of the lower mantle, the results of Rudolph *et al*.^17^ also are consistent with our results (Fig. 4b). In that case only the variations can be compared since Rudolph *et al*.^17^ propose only relative viscosity profiles. However, one can see on Fig. 4b that the range of viscosity corresponding to the hill that they propose at 1000–1500 km depth can well be described within the variability of our model.

## Conclusions and Perspectives

In a framework where the rheology of the lower mantle is primarily constrained by pure climb creep, ultimately controlled by diffusion, our progress on understanding the viscosity of this region will be controlled by our knowledge of this key parameter. Experimental data on diffusion in bridgmanite are limited, due to the difficulties of performing diffusion experiments at high pressure and also because diffusion appears to be very slow in bridgmanite, making the measurement of diffusion profiles a very challenging task. Figure 4 shows that the viscosity profiles based upon geophysical observations and their variation with depth fall wholly in the range of conditions (stresses, vacancy concentrations) that can be anticipated for the mantle using the present approach. Indeed, in the lower mantle, bridgmanite may exhibit radial and lateral variations (at different scales) in chemistry, not captured in PREM^64^, which may affect vacancy concentrations, the shear modulus and the diffusivity. In this paper, we kept the discussion in the very simple framework of a 1D viscosity profile throughout the lower mantle. The lower mantle is, however, expected to present heterogeneities at different scales. For example, Ballmer *et al*.^69^ have shown that large scale heterogeneities such as the BEAMS (bridgmanite−enriched ancient mantle structures) can survive (and even organize) mantle convection. Recently, Shim *et al*.^70^ suggested that iron partitioning between bridgmanite and ferropericlase could be significantly affected by large changes in the oxidation state of iron in bridgmanite. They proposed a lowering of the total iron content in bridgmanite in the mid–mantle that they relate to viscosity changes through scaling laws such as the homologous temperature relation. In this context, more detailed predictions could be made by relating chemistry and oxidation state to vacancy concentrations. This would require, among other things, a better understanding of point defect chemistry in bridgmanite. To the best of our knowledge, the only detailed theoretical study of point defect chemistry is the one of Hirsch & Shankland^71^ for Fe−bearing bridgmanite. It must be recognized that, although much progress has been made on this front, the actual species of iron in bridgmanite (oxidization state, spin state, and associated crystallographic sites) are still not well constrained. Moreover, the study of Hirsch & Shankland^71^ did not consider the presence of aluminium. Efforts to better understand the point defects that control creep in bridgmanite and their concentrations in the lower mantle should be a priority in the near future.

## Supplementary information

Supplementary Material
