# Supplementary material for: The role of diffusion-driven pure climb creep on the rheology of bridgmanite under lower mantle conditions
Source: Sci Rep. 2019 Feb 14;9:2053. doi: 10.1038/s41598-018-38449-8 (PMC6376055; doi:10.1038/s41598-018-38449-8)
Supplement: Supplementary file 1 — Supplementary Material [file 41598_2018_38449_MOESM1_ESM.docx]

**SUPPLEMENARY MATERIAL OF THE PAPER:**

**The role of diffusion-driven pure climb creep on the rheology of bridgmanite under lower mantle conditions**

Riccardo Reali^1*^, James A. Van Orman^2^, Jeffrey S. Pigott^2,3^, Jennifer M. Jackson^4^, Francesca Boioli^1,5^, Philippe Carrez^1^, Patrick Cordier^1^

^1^Univ. Lille, CNRS, INRA, ENSCL, UMR 8207 − UMET − Unité Matériaux et Transformations, Lille F-59000, France

^2^Department of Earth, Environmental, and Planetary Sciences − Case Western Reserve University, 10900 Euclid Avenue, Cleveland, OH, 44106, U.S.A.

^3^Present address: Shock and Detonation Physics (M-9), Los Alamos National Laboratory, Los Alamos, NM, 87545, U.S.A.

^4^Seismological Laboratory, Division of Geological and Planetary Sciences − California Institute of Technology, Pasadena, CA, 91125, U.S.A.

^5^Present address: LEM, UMR 104 CNRS/ONERA, Chatillon 92320, France

Submitted to: Scientific Reports

**^*^Corresponding author:**

Riccardo Reali

University of Lille

Unité Matériaux et Transformations (UMET) – CNRS UMR 8207 – Bat C6

59655 Villeneuve d’Ascq Cedex, France

Tel: +33771236304

E-mail: riccardo.reali@univ-lille.fr


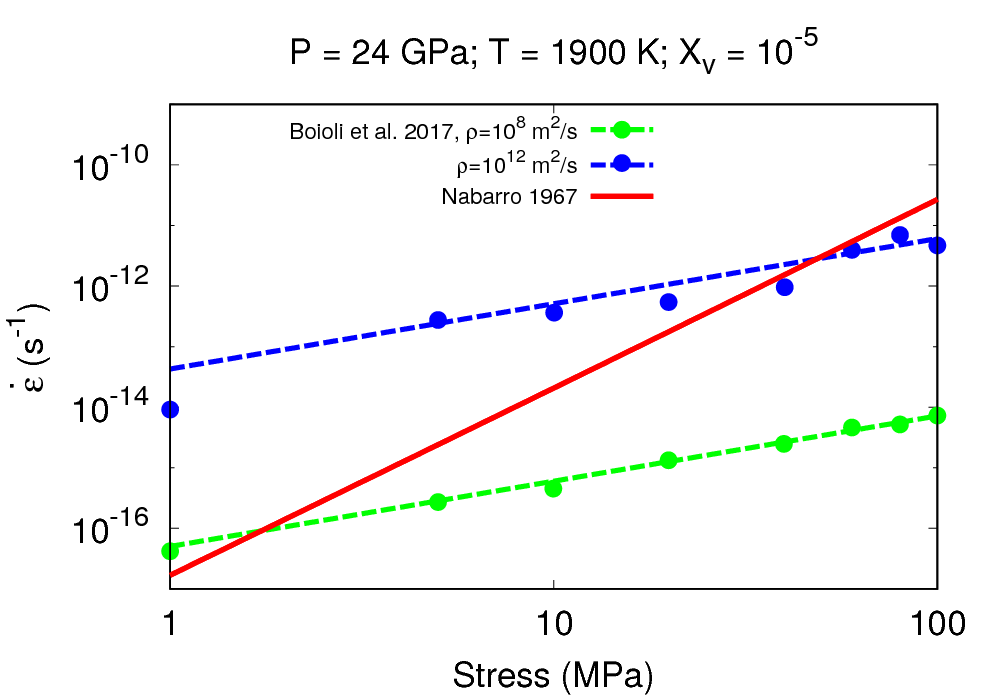


**Supplementary Figure 1. Comparison between data from pure climb numerical modeling obtained with Dislocation Dynamics^1^ and the Nabarro^2^ model.** The Nabarro^2^ creep model (red line) reproduces well the results obtained by 2.5D Dislocation Dynamics simulations of pure climb creep (green and blue dots and relative fit lines). The *PT* conditions as well as the vacancy concentration *X_v_* are the same for the current model and the numerical simulations results and are specified in the title of the figure. The main difference between these approaches is related to dislocation density. In fact, in the present study we implement a creep model^2^ that is formulated accounting for an equilibrium dislocation density that depends on the applied stress and leads to a steady−state creep condition (see the main text for further details). On the other hand, different values of the initial dislocation density have been used as input parameter in the 2.5D−DD model. In particular, the initial dislocation density was set equal to 10^8^ and 10^12^ m^-2^(green and blue curves in Supplementary Fig. 1, respectively).

A unification of these results can be achieved by considering the well−established Taylor equation that scales the dislocation density *ρ* as function of the applied stress *σ*:

$\rho=\left( \frac{\sigma}{\alpha b\mu} \right)^{2}$ (S1)

where α is the adimensional Taylor coefficient, *b* is the Burgers vector and *μ* the shear modulus. α does not appear in the pure climb creep formulation^2^ and is therefore set to be 1, while at these conditions *b* was estimated equal to 4.65^3^ Å and *μ* to 167^4^ GPa. With equation (S1) it is easily calculated that, for stresses of 1 and 100 MPa, the relative dislocation density is equal to ~10^8^ and 10^12^ m^-2^ respectively. This is in perfect agreement with the 2.5D Dislocation Dynamics data^1^, reported here with the green and blue curves, obtained for a fixed dislocation density of 10^8^ and 10^12^ m^-2^, respectively. The present model^2^, in fact, reproduces comparable strain rates (*i.e.*, the red line crosses the green and blue lines) at identical dislocation densities and stresses of 1 and 100 MPa.

**
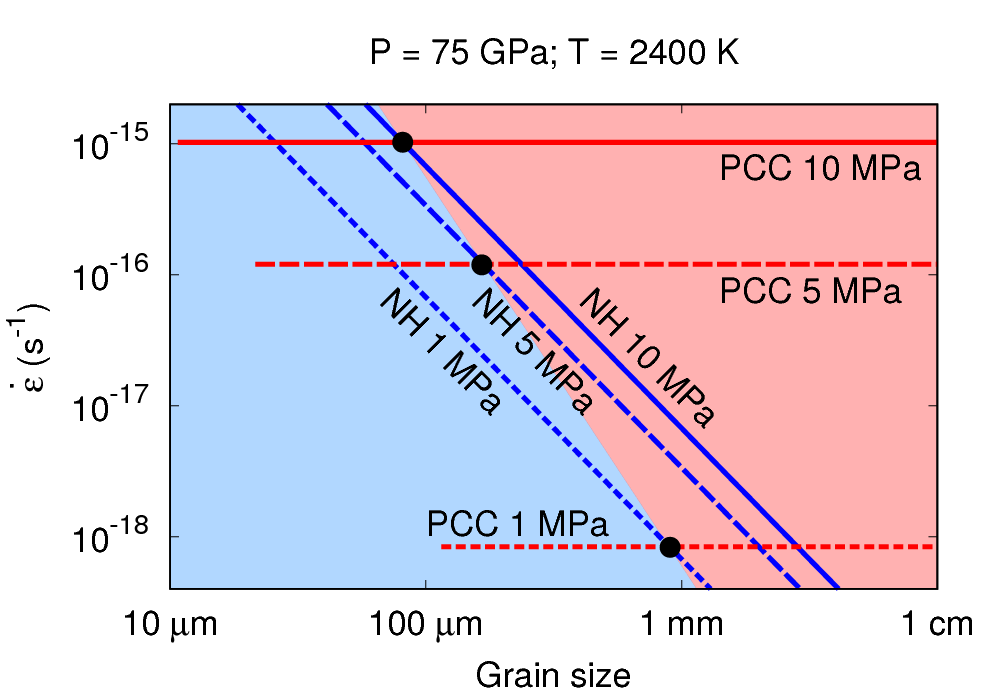
**

**Supplementary Figure 2.** Deformation map (strain rate vs. grain size) at 75 GPa, 2400 K comparing pure climb creep (PCC) and Nabarro–Herring (NH) mechanisms.

**
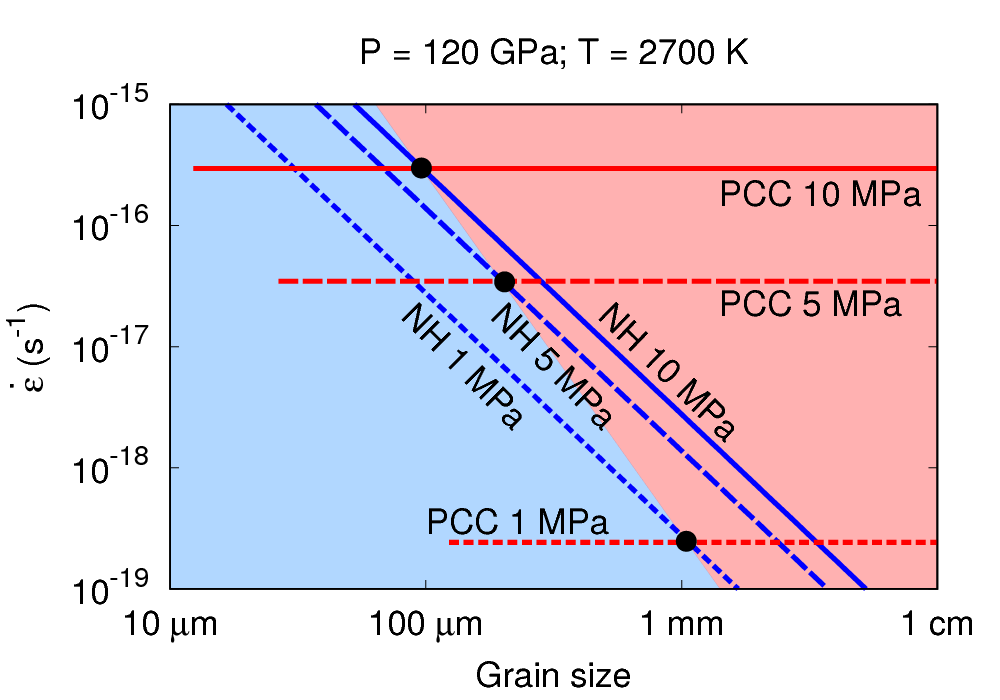
**

**Supplementary Figure 3.** Deformation map (strain rate vs. grain size) at 120 GPa, 2700 K comparing pure climb creep (PCC) and Nabarro–Herring (NH) mechanisms.

| *P* (GPa) | *Depth* (km) | *T* (K) | $\dot{\varepsilon}$ (s^-1^) | *η* (Pa∙s) |
| --- | --- | --- | --- | --- |
| 30 | 809 | 1992 | 1.3 ∙ 10^-11^ | 7.9 ∙ 10^17^ |
| 60 | 1457 | 2256 | 1.9 ∙ 10^-12^ | 5.1 ∙ 10^18^ |
| 90 | 2060 | 2487 | 6.2 ∙ 10^-13^ | 1.6 ∙ 10^19^ |
| 120 | 2617 | 2689 | 3.0 ∙ 10^-13^ | 3.4 ∙ 10^19^ |

**Supplementary Table 1.** Estimate of the strain rates $\dot{\varepsilon}$ and viscosities *η* at different pressures along the geotherm using the creep model^2^ displayed in equation (3). Here the vacancy concentration *X_v_* and the deviatoric stress are equal to 10^-2^ and 10 MPa, respectively.

| *P* (GPa) | *Depth* (km) | *T* (K) | $\dot{\varepsilon}$ (s^-1^) | *η* (Pa∙s) |
| --- | --- | --- | --- | --- |
| 30 | 809 | 1992 | 1.0 ∙ 10^-18^ | 9.8 ∙ 10^23^ |
| 60 | 1457 | 2256 | 1.6 ∙ 10^-19^ | 6.3 ∙ 10^24^ |
| 90 | 2060 | 2487 | 5.1 ∙ 10^-20^ | 2.0 ∙ 10^25^ |
| 120 | 2617 | 2689 | 2.4 ∙ 10^-20^ | 4.1 ∙ 10^25^ |

**Supplementary Table 2.** Estimate of the strain rates $\dot{\varepsilon}$ and viscosities *η* at different pressures along the geotherm using the creep model^2^ displayed in equation (3). Here the vacancy concentration *X_v_* and the deviatoric stress are equal to 10^-6^ and 1 MPa, respectively.

| *P* (GPa) | *Depth* (km) | *T* (K) | $\dot{\varepsilon}$ (s^-1^) | *η* (Pa∙s) |
| --- | --- | --- | --- | --- |
| 30 | 809 | 1992 | 1.3 ∙ 10^-15^ | 7.9 ∙ 10^21^ |
| 60 | 1457 | 2256 | 1.9 ∙ 10^-16^ | 5.1 ∙ 10^22^ |
| 90 | 2060 | 2487 | 6.2 ∙ 10^-17^ | 1.6 ∙ 10^23^ |
| 120 | 2617 | 2689 | 3.0 ∙ 10^-17^ | 3.4 ∙ 10^23^ |

**Supplementary Table 3.** Estimate of the strain rates $\dot{\varepsilon}$ and viscosities *η* at different pressures along the geotherm using the creep model^2^ displayed in equation (3). Here the vacancy concentration *X_v_* and the deviatoric stress are equal to 10^-6^ and 10 MPa, respectively.

**Supplementary references**

1. Boioli, F. *et al.* Pure climb creep mechanism drives flow in the Earth’s lower mantle. *Sci. Adv.* **3(3)**, e1601958 (2017).

2. Nabarro, F. R. N. Steady-state diffusional creep. *Philos. Mag. A* **16**, 231-237 (1967).

3. Kraych, A., Carrez, P. & Cordier, P. On dislocation glide in MgSiO_3_ bridgmanite, *Earth Planet. Sc. Lett.* **452**, 60-68 (2016).

4. Dziewonski, A. M. & Anderson, D. L. Preliminary reference Earth model. *Phys. Earth Planet.Int.* **25**, 297-356 (1981).
